# Evidence for cellular and solute passage between the brain and skull bone marrow across meninges: A systematic review

**DOI:** 10.1177/0271678X251316392

**Published:** 2025-01-25

**Authors:** Helena Eide Therkelsen, Rune Enger, Per Kristian Eide, Geir Ringstad

**Affiliations:** 1 Faculty of Medicine, University of Oslo, Oslo, Norway; 2 GliaLab and the Letten Centre, Division of Anatomy, Department of Molecular Medicine, Institute of Basic Medical Sciences, University of Oslo, Oslo, Norway; 3 KG Jebsen Centre for Brain Fluid Research, University of Oslo, Oslo, Norway; 4 Department of Neurosurgery, Oslo University Hospital-Rikshospitalet, Oslo, Norway; 5 Institute of Clinical Medicine, Faculty of Medicine, University of Oslo, Oslo, Norway; 6 Department of Radiology, Oslo University Hospital-Rikshospitalet, Oslo, Norway; 7 Department of Geriatrics and Internal Medicine, Sorlandet Hospital, Arendal, Norway

**Keywords:** Brain meninges, cerebrospinal fluid, immune cells, skull bone marrow, skull channels

## Abstract

A potential two-way passage of cells and substances between the brain and skull bone marrow may open for new insights into neurological disease. The arachnoid membrane was traditionally considered to restrict cells and larger molecules in CSF from entering the dura and bone marrow directly. However, new data on exchange between brain and skull bone marrow have recently emerged. Here, we conducted a systematic literature to answer the question: What is the current evidence regarding the movement of cells and molecules between the brain and skull bone marrow, spanning CSF and meninges? We excluded studies related to head or skull trauma, cranial fractures or defects, cancer invasion, CSF leakage, spontaneous intracranial hypotension, spinal dura mater, and studies solely focusing on meningeal lymphatic vessels or the passage of substances from CSF to meningeal lymphatic vessels. The review identified 16 studies that provide evidence of communication between the brain, meninges and skull bone marrow. Cells (such as B and T cells and neutrophils), bacteria, and substances (tracers, drug compounds) have been reported to pass between the brain and skull bone. However, most studies are performed in rodents, emphasizing the need for translation to humans.

## Introduction

Leveraging groundbreaking discoveries made in 2015,^1,2^ which unveiled the presence of functional meningeal lymphatic vessels capable of transporting substances from the cerebrospinal fluid (CSF) to extra-cranial lymph nodes, a profound resurgence of interest has emerged regarding the organization and functional properties of the brain meninges and skull bone marrow. There is a growing interest how the brain may interact via CSF with the peripheral and central immune system at brain borders (subarachnoid CSF, brain meninges and skull bone marrow), and its role in immunosurveillance of the central nervous system (CNS) and clearance of waste substances from the CSF. At the same time, there is an ongoing debate about the anatomical and molecular organization of CNS barriers, the compartmentalization of the subarachnoid space, and the extent to which meninges function as impermeable or semipermeable barriers for cells and solutes.^3–6^

Traditionally, the arachnoid and dural meninges were deemed largely impermeable barriers to passage of substances and cells.^4,7,8^ However, experimental studies have reported the passage of immune cells from the CSF to the meninges,^1,2,9^ or vice versa,^10,11^ with CSF governing immune cell migration from skull bone marrow to the dura.^12^ Critics argue that these studies may overlook considerations of the properties of the arachnoid mater, forming a barrier between the subarachnoid space and the dura mater.^4^

**Figure 1.**
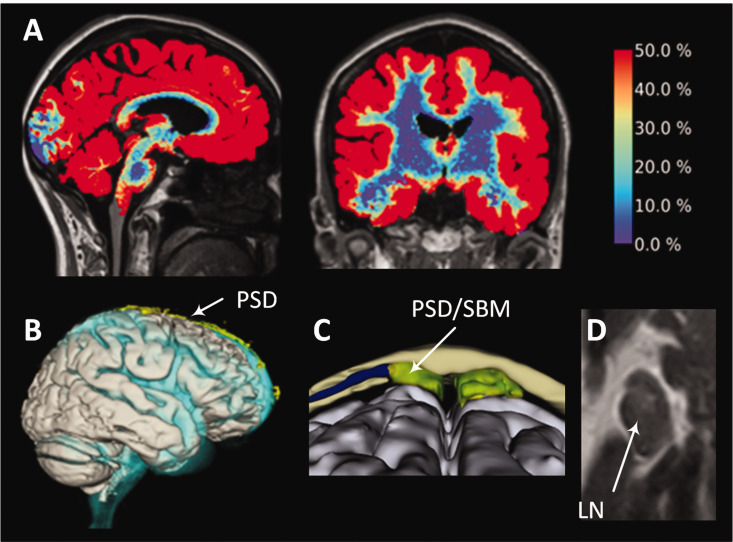
An intrathecal tracer distributes within brain, parasagittal dura, skull bone marrow and extracranial lymph nodes in humans. Following the intrathecal injection of an MRI contrast agent (gadobutrol, with a molecular weight of 604 Da) as a cerebrospinal fluid (CSF) tracer, the distribution is observed in (A) the entire brain^13^ (color bar on the right indicating the percentage increase in tracer enrichment after about 24 hours). Additionally, the tracer disperses within (B) the parasagittal dura (PSD) near the superior sagittal sinus,^14^ (C) PSD within skull bone marrow (SBM),^15^ and tracer enrichment was seen within (D) extracranial lymph nodes (LN).^16^ These tracer studies consistently reveal that the peak tracer enrichment in various compartments typically occurs around 24 hours, suggesting a prolonged process.

From a clinical perspective, human studies utilizing intrathecal magnetic resonance imaging (MRI) contrast agent (gadobutrol) as a CSF tracer have revealed brain-wide extravascular tracer enrichment,^13^ as well as tracer enrichment in parasagittal dura along the superior sagittal sinus,^14^ skull bone marrow,^15^ and extracranial lymph nodes^16^ (see Figure 1). These observations suggest the passage of the tracer from the subarachnoid CSF space across the meninges to the skull bone marrow and extracranial lymph nodes, challenging the previous notion of arachnoid impermeability. Critics, on the other hand, may contend that tracer enrichment in various tissues after intrathecal administration is due to recirculation of the CSF tracer through the vascular compartment. An argument against this is that the tracer is administered in a low dose (0.25–0.50 mmol), with very limited passage into the vasculature over the intact blood-brain-barrier, and tracer concentrations in blood at µmol level^17^ are unlikely detected at MRI.

Given the present controversy, this study addressed the question about the transport of cells and solutes between the brain and the skull bone marrow across the meninges. We systematically reviewed the literature, asking: What is the current evidence for passage of molecules and cells between the brain, CSF of subarachnoid space, brain meninges, and the skull bone marrow? For this purpose, we excluded studies addressing traumatic head injuries, skull fractures, or spontaneous cerebrospinal fluid (CSF) leaks, all potentially causing abnormal passage of cells and molecules between the brain and skull bone marrow.

## Materials and methods

### Data sources

A systematic search was conducted utilizing the following bibliographic databases: EMBASE (Ovid), MEDLINE (Ovid), The Cochrane Library. The search was limited to English articles and included publications from any date. Unpublished studies, gray literature (e.g., reports, white papers, and newsletters), abstracts and conference presentations were excluded.

We formulated the following research question initiating the literature search: *What is the current evidence for passage of molecules and cells between brain, CSF and skull bone marrow across dura mater in the absence of traumatic or non-traumatic dura leakage?*

Inclusion criteria were: English language, human and animal studies, and studies addressing the crosstalk of cells and substances between the brain, CSF, and cranial structures across the meninges.

Exclusion criteria were: Head and/or skull trauma, head injury, post-traumatic brain injury leakage, cranial fracture, cranial and/or calvarial defect, cranial defect repair, dura-bone formation, dural impact on bone formation and healing, cranial fracture healing, cancer invasion, CSF/dura leakage, spontaneous intracranial hypotension, spinal dura, and vertebral lymphatic vessels. Studies addressing the passage of cells between anatomic structures during embryogenesis were not considered. Additionally, studies solely focusing on meningeal lymphatic vessels or the role of meningeal lymphatic drainage failure in neurological disease were excluded.

The full search strategy is presented in the Supplementary Table 1.

### Study selection

The search team comprised all four authors. The first author (HET) conducted the initial search and selection, which was subsequently reviewed by the other authors RE, PKE, and GR. A digital library was established using EndNote and shared among the authors.

### Data extraction

The data extracted included 1) Methods used (including species studied, method for estimating crosstalk between the brain, CSF, dural meninges and skull bone marrow, 2) Anatomical evidence for cross-talk between CSF and skull bone marrow at meninges. 3) Functional evidence for cross-talk, i.e. evidence for passage of either cells or molecules between the brain, CSF and skull bone marrow across the meninges. We distinguished between animal and human studies.

## Results

### Study selection

**Figure 2.**
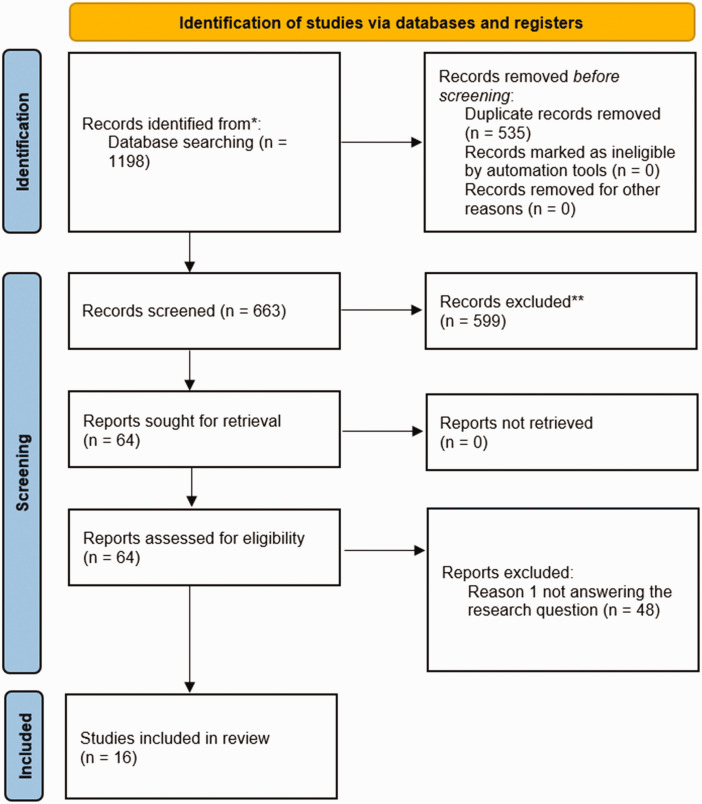
PRISMA. Flow diagram of included studies.^18^

Figure 2 presents a PRISMA flow diagram^18^ of the review (http://www.prisma-statement.org/PRISMAStatement/PRISMAStatement.aspx). The final search was conducted on January 11^th^, 2024. After eliminating duplicates, 663 publications were initially identified. Following the application of criteria, 647 publications were excluded, resulting in 16 selected publications. Among these, 10 were animal studies, 3 were human studies, and 3 encompassed both animals and humans. The search strategy employed in Medline, Embase, and the Cochrane Library is outlined in Supplementary Table 1, and the sixteen included references are provided in Supplementary Table 2.

**Figure 3.**
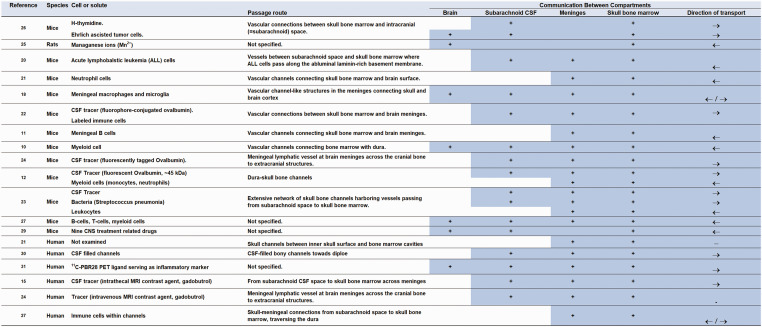
Overview of studies included in the systematic review. The literature review differentiated between animal and huma studies. → indicates transport in direction from subarachnoid space towards skull bone marrow. ← indicates transport in direction from skull bone marrow towards subarachnoid space.

Results from animal studies are detailed in Table 1, while human study outcomes are presented in Table 2. Additionally, an overview of the results is depicted in Figure 3.

**Table 1.** Evidence from animal studies of cellular and solute passage between brain, CSF and skull bone marrow across meninges.

Reference	Methodology	Anatomical evidence	Functional evidence	
			Cellular transport	Molecular transport
Srebro et al. 1993^24^	Species: MiceH-thymidine injected intrathecally in mice. Tumor cells injected into the subarachnoid space, brain ventricles or brain parenchyma in mice. Histological sections of brain: Brain with the sphenoid bone fixated, sectioned and stained or auto radiographed and left unstained.	Intrathecal injected H-thymidine passed unto the sphenoid bone marrow from subarachnoid space, where it labeled leukocytesTumor cells injected penetrated into vessels and some were found in dilated sinusoids of the skull bone marrowHistological sections of brain with attached sphenoid bone show vascular connections between what the authors denote “intracranial space” and bone marrow; Small vessels derived from the meninges penetrate into sphenoid bone marrow through small openings/canals in the sphenoid bone. Canals in the external lamina of sphenoid bone connects the “intracranial space” with the bone marrow space.	Tumor cells	Tritiated H-thymidine (molecule)
Atanasijei et al. 2017^26^	Species: RatsExperimental study on rats: The skull bone was exposed, and MnCl_2_ solution applied tothe skull. MRI was utilized to detect Manganese diffusion into the brain, showing detectable tissue enhancementThe local concentration of Manganese was quantified in the brain after transcranial manganese application. The authors investigated the effectiveness of Manganese passage though different regions of the skullOther salts like calcium were added to solution of MnCl_2_	Manganese ions (Mn^2+^) was found to diffuse through rat skull bone to meninges, and then manganese penetrated the meninges to cortical tissueConcentrated solutions of MnCl_2_, have high osmolality and can pass through intact rat skull bone. Solutions of lower Manganese concertation were only able to diffuse through the skull when Calcium salts were added. Previously undetectable applied Manganese concentrations became detectable in the brain tissue	Not observed	Manganese ions (Mn^2+^)
Yao et al. 2018^20^	Species: MiceIn-vitro study Histologic cross-sectionsImmunohistochemical staining of channels.	Acute lymphoblastic leukemia (ALL) cells migrate into the subarachnoid space along bridging vessels that pass directly between the skull bone marrow and the subarachnoid space. The basement membrane of the bridging vessels was enriched with laminin. The cells migrated along the laminin-enriched extracellular matrix of emissary bridging vessels (i.e., laminin receptor α6 integrin-dependent migratory pathways) that pass between calvarial bone marrow and subarachnoid space, Evidence for existence of bony vascular channels that connect the bone marrow and the meninges. These channels contain vessels that connect the skull bone marrow with the subarachnoid space.	Acute lymphoblastic leukemia cells found in transit surrounding bridging vessels between skull bone marrow and subarachnoid space, traveling along the external surface of vessels	Not specified
Herisson et al. 2018^21^	Species: MiceIn vivo cell labeling of bone marrow cells in the mouse skull and tibia. Cell tracking by flow cytometry. Murine models of acute inflammation including ischemic stroke etc. Confocal microscopy of skull-dura interface including of channels connecting the skull marrow to dura. Microscopy of the interior surface of the harvested skull, submerged in an organ bath to observe channels. Imaged 4-week mice in vivo, to determine cell traffic in the channels in vivo. Electron microscopy of channels.	Observed channels that connect the skull bone marrow cavities with the dura mater, traversing through the inner skull cortex when studying skull-brain intersection by confocal microscopyDirect microscopic vascular channelsElectron microscopy confirmed channels lined with endothelium, the channel lumen connects with vasculature in the dura mater. Neutrophils exited channels more frequently in skulls harvested from mice with brain inflammation.	Neutrophils migration through bone marrow channels. Neutrophils exited blood vessels in the dura.	Not specified
Wang et al. 2019^19^	Species: Mice Light-sheet microscopy LSM) enabled non-invasive in vivo 3 D imaging through mouse head of disease models in live mice. Non-invasive in vivo light-sheet microscopy (LSM) of mouse traumatic brain injury models. Meningeal macrophages and microglia labeled with fluorescents	Observed vascular channel-like structures in the meninges connecting the brain cortex and skull bone. Channels resolved by non-invasive LSM imaging in-vivo	Labeled meningeal macrophages and microglia migration to the traumatic brain injury site.	Not specified
Cai et al. 2019^22^	Species: MiceLight sheet microscopy (LSM) imaging of transparent mice by vDisco-nanoboosting method: An immunolabeling technology to enhance signal of fluorescent proteins using nanobody-labeling-methodA technique that allows light-sheet microscopy scanning of transparent mice/panoptic imaging of mice and quantification of subcellular details in tissues through intact bone, allow imaging of immune celled and details of meningeal vessels. Middle-cerebral-artery occlusion (MCAO) model of strokeCSF tracer injected into cisterna magna in mice to study vascular connection, in addition to another vessel tracer	Observed vascular connections between the skull bone marrow and the surface of the brain meninges, named “short skull meningo-connections (SMCs) Observed tracer labeled meningeal vessels that extended into the skull	Observed that the vascular connections occasionally contained cells. Labeled immune cells were observed in the vascular connections after induced stroke (MCAO model)	CSF tracer injected into the cisterna magna passed into meningeal vessels that extended into the skull.
Brioschi et al. 2021^11^	Species: Mice Investigated origin and dynamics of meningeal B cells. The mouse meninges were studied using single-cell RNA- sequencing, cytometry by time of flight, single-cell B cell receptor sequencing. Verified their findings by flow cytometry and confocal imaging. Determined origin of B cells in the meninges by bone marrow transplantation and parabiosis experiments.	Meningeal B cells originate from the calvaria and migrate to the meninges through specialized channels; skull vascular channels, traversing the inner skull bone. B cells complete their development locally in the meninges. Early subsets of B cell, that are normally present in the bone marrow, are also found in the meninges under hemostasis, while circulating B cells minimally infiltrate the mouse meninges under hemostasis.	B cells (lymphocytes)	Not specified
Cugurra et al. 2021^10^	Species: MiceAuthors studied sources of myeloid cell infiltrates and how they access brain parenchyma using a mouse model of CNS disease. Using several approaches, including calvarial bone-flap transplantation, selective irradiation regimens, and bone marrow transfers. Using various CNS injury models including spinal cord injury, optic nerve crush. Using RNA sequencing to study roles of monocytes derived from blood and from skull bone marrow.	Myeloid cells not derived from blood had originated from skull and vertebral bone marrow. Bone marrow next to the brain supply monocytes to the meninges both under homeostasis and after CNS injury: Under hemostasis the dural meninges host monocytes derived from the local skull bone marrow. During CNS injury, skull bone marrow contributes to the infiltration of myeloid cells into brain parenchyma/ skull bone marrow derived monocytes can infiltrate the brain parenchyma. Channels connecting the skull bone marrow and dura allow a route for the migration of myeloid cells from local bone marrow to dural meninges.	Myeloid cells from skull- and vertebral bone marrow to brain meninges and brain parenchyma	Not specified
Jacob et al. 2022^25^	Species: Mice Tracked lymphatic drainage in dura mater using intra-cerebrospinal fluid injections of Ovalbumin tracer. Stereomicroscope imagining +Postmortem light sheet fluorescence microscopy (LSFM) allowed 3 D imaging/mapping of cranial CSF outflow pathways in mice, after injection of fluorescently tagged Ovalbumin	Tracer uptake by meningeal lymphatic vessels in the calvariaMeningeal lymphatic vessels penetrating the skull through bilateral foramina in the skull base	Not documented	Fluorescently tagged ovalbumin.
Mazzitelli et al. 2022^12^	Species: MiceCSF tracer (Ovalbumin) injected to cisterna magna and efflux to skull bone marrow studied. Examined skull base and skull base bone marrow. Used two-photon microscopy to observe CSF tracer in dorsal skull bone marrow. To confirm that CSF interact with cells in calvarial bone marrow they assessed the accumulation of Ovalbumin signal in macrophages and observed uptake in skull after injection in cisterna magna. To show that CSF interacts with other cells in bone marrow spaces they assessed labeling of hematopoietic stem cells by flow cytometry after injection of antibody	Uptake of CSF tracer along dural sinuses and uptake of tracer along perivascular spaces within dura-skull channels and in the bone marrowObserved tracer accumulation in bone marrow of skull after injection of tracer into cisterna magnaOssified skull bone channels from tabula interna towards dura mater to skull bone marrow. One hour after injection antibody in cisterna magna 99 % of LSK cells (type of hematopoietic stem cells) in the skull bone marrow were labeled. Injection of component of gram-negative bacteria into CSF of mouse lead to the expansion of skull bone marrow myeloid progenitors	Myeloid cells (monocytes, neutrophils) travels from skull bone marrow to the dura in response to CSF-derived signals, here spinal cord injury. Spinal cord injury caused an increase in myeloid cells in dural meninges and a decrease of myeloid cells in overlying skull bone marrow.	CSF tracer (Ovalbumin) pass from SAS to skull bone marrow after injection to cisterna magna. The tracer administered to SAS passed to dura along dural vein sinuses and then along perivascular conduits within skull channels to the bone marrow space.
Pulous et al. 2022^23^	Species: MiceComputed tomography (CT) of skull bone: Spatial organization of skull bone channelsTwo-photon microscopy of bone-marrow: Passage of fluorescent tracer after injection into cisterna magna. Transmission electron microscopy (TEM) of skull marrow cavity and skull channels: Ex-vivo visualization of CSF tracer distributionStreptococcus pneumonia was administered to cisterna magna of mice and whole-body bioluminescence imaging performed to analyze bacterial propagation over time. Green fluorescent protein expressing S. pneumonia was injected nto cisterna magna and GFP+ bacterial colonies were observed in skull marrow with confocal microscopy in mice with meningitis	Data show that CSF travel from subarachnoid space (SAS) into dura along the perivascular space of dural blood vessels, and then perivascular through skull channels to the skull bone marrow cavities. The study visualized injected tracer in skull channels, the signal surrounded the blood vessel, all the way from the dural opening of the channel to the bone marrow The channels have a dural opening and go into the skull bone marrow cavity, they link skull bone marrow with dura mater. Data show that CSF passes perivascularly from the SAS along a subset of dural blood vessels that goes into the skull bone marrow through bone channels. CSF passes through the perivascular space of skull channels into the cranial bone marrow.	In mice with meningitis, observed passage of the streptococcus pneumonia bacteria to the skull bone marrow trough skull channels form meninges that induces proliferation of hematopoietic progenitor cells in calvarial bone marrowMigration of leukocytes (myeloid cells, lymphocytes) from skull bone to dural meninges	CSF tracer (Ovalbumin, Dextran,) can pass into skull bone marrow after injection into cisterna magna. Intracisternal injection reveled a similar perivascular appearance for tracers with molecular weights 66 kDa to 2,000 kDa.
Kolabas et al. 2023^27^	Species: MiceMCAo as a model for stroke in mice Two-photon imaging of the skull after strokeKiKGR mouse model to study immune cells in skull marrow and brain.	Exact anatomical route not specified.	Detected RFP+ B, T and myeloid cells in ipsilateral brain 1 h and 6 h after photo conversion, indicating that immune cells from skull marrow are recruited to the brain after injury.	
Kang et al. 2023^29^	Species: MiceAim: Evaluate drug penetration into the brain by intraosseous administration into the skull. Type study: Quantitative pharmacokinetic analysis with 8 CNS treatment related compounds and a control. How: 9 compounds (eg. Risperidone, donepezil) was applied over thinned mouse skulls and the compound entry level in the brain was measured and compared to that after systemic administrationPharmacokinetic and in-vivo brain uptake study after ICO and IV administration	Exact anatomical route not specified.	Not performed	The tested compounds reached the brain tissue several tens-to-hundred times higher by intracalvariosseous application than by systemic application.

**Table 2.** Evidence from human studies of cellular and solute passage between brain, CSF and skull bone marrow across meninges.

Reference	Methodology	Anatomical evidence	Functional evidence	
			Cellular transport	Molecular transport
Herisson, et al. 2018^21^	Micro Computed Tomography (microCT) of craniotomy specimens that were acquired during decompression surgery in patients.	Identified skull channels connecting the surface of the inner skull cortex with bone marrow cavities.	Not examined.	Not specified
Tsutsumi et al. 2019^30^	Human research: 358 patients with neurological symptoms such as headaches, dizziness, vertigo, tinnitus, hearing disturbances, hemisensory disturbances, seizuresImaging: T2-weighted MRI sequences involving the whole cranial base and calvarial vault	Identified CSF-filled channels in the anterolateral base of the middle fossa and diploe of the pterional region. The CSF-filled spaces were continuous with the subarachnoid spaces. High-intensity structure identified in the anterolateral base of the middle fossa and diploe of the pterional region was clearly continues with the intracranial subarachnoid space at its medial-most part.	Not observed	CSF-filled.
Hadjikhani et al. 2020^31^	Aim: Examine the upregulation of an inflammatory marker in the meninges and skull bone in migraine patients with auraImaging method: ^11^C-PBR28 PET/MRI of a ligand that detects activated inflammatory cells, in human migraine patients with visual auraMeasured tracer uptake in four regions of interest consisting of the meninges plus the overlying skull bone Compared results to healthy controls and chronic pain patients	The anatomical transport route was not specified. Detected a significantly higher mean tracer uptake in the meninges (dura) and skull bone overlying the occipital cortex in patients with migraine and aura than control groups. Enhanced PET signal / PET signal enhancement in meninges and calvarium overlying occipital cortex, in other words an elevated inflammatory signal found in meninges and skull bone.	Not observed	Inflammatory marker (^11^C-PBR28) (Tracer)
Ringstad & Eide, 2022^15^	In-vivo study in humans with tentative CSF disorders. Imaging method: Intrathecal contrast-enhanced MRI to measure tracer enrichment as increase in MRI T1 signal over time after its intrathecal injection. Tracer: Gadobutrol, an MRI contrast agent, served as CSF tracer.	The anatomical transport route was not specified. CSF tracer enrichment assessed by increase in normalized MRI T1 signal in CSF, parasagittal dura and skull bone marrow suggested CSF tracer passage from CSF of SAS, across dura to skull bone marrow in patients with tentative CSF disorders.	Not observed	Gadobutrol (CSF tracer) - a hydrophilic molecule with molecular weight 620Da
Jacob et al. 2022^25^	In-vivo study in humans with neurological disordersImaging method: Used MRI (T1 and FLAIR sequences) to depict lymphatic drainage in dura mater of patients after systemic injection of the MRI contrast agentTracer: Gadobutrol, an MRI contrast agent Generated a 3D map of different gadobutrol flow circuits	Evidence of transcranial vessels that penetrate the skull and join subcutaneous lymphatic and venous vasculature. Study found perforating venules and slow-flow channels, parallel to superior sagittal sinus, crossing the skull and connecting to superficial intracalvaria and subcutaneous vasculature, a link between dura and calvarial bone (In a patient with Gorham-Stout disease there was seen a strong enhancement of slow-flow gadobutrol in the vanished parietal bone and the associated dural area)	Not observed	Intravenous MRI contrast agent (adobutrol).
Kolabas et al. 2023^27^	Human post-mortem skull + dura mater samples from human skullsTissue clearing and light-sheet fluorescent imaging. Immunofluorescence to label myeloid cells and macrophages. Bright field imaging of sections of human skull and immunohistochemistryPerformed scanning electron microscopy on skull + dura materOptical cleaning of the skull, meninges, brain specimen	Characterized skull-meninges-connections (SMCs) at the cellular level that connects the subarachnoid space with the skull bone marrow traversing dura mater.Human SMCs transverse the dura mater, opening to the subdural space underneath to arachnoid granulations	The skull-meninges-connection showed immune cells within, in addition to a fibroblastic cell layer	

### Animal studies

#### Anatomical evidence

Regarding the anatomical route for potential cell and solute passage, studies in rodents have demonstrated skull-meningeal channels, acting as bridges between meninges, the subarachnoid space, and skull bone marrow.^10–12,19–24^ Moreover, channels in the calvarial bone have been shown to harbor vessels crossing from the dural meninges to the skull bone marrow.^20,23,24^ Pulous et al.^23^ estimated that in an adult mouse, over 1,000 bone channels reach into the cranial vault. Experimental evidence suggests that cells and solutes in the CSF pass outside vessels traversing the bone marrow cavities, meninges, and the subarachnoid space.^12,19,20,23,24^ Notably, CSF tracers with molecular weights ranging from 66 kDa to 2,000 kDa appeared along a subset of dural vessels, providing evidence of passage of large molecules between subarachnoid CSF spaces and skull bone marrow through an extensive skull channel network.^23^ Among the identified studies, eight^10–12,21–23,25,26^ provided evidence for cell and solute passage between the meninges and skull bone, five^12,20,23–25^ between the subarachnoid space and the skull bone, and three between the brain cortex, meninges, and skull bone.^10,24,26^

To the best of our knowledge, a study in 1993^24^ first demonstrated meningeal vessels passing through openings or channels in the skull bone that expand into sinusoids within the bone marrow cavity. In this study, histological images of the brain with attached sphenoid bone showed the existence of vascular connections or canals between what the authors denote as “intracranial” space and the skull bone marrow.^24^ However, the microvessels claimed to bridge the subarachnoid space and skull bone marrow were not directly visualized. More recently, others utilized a light-sheet microscopy methodology to identify “vascular-channel-like structures” in the meninges connecting the brain cortex with the skull bone,^19^ and meningeal lymphatic vessels penetrating the skull via openings at the skull base.^25^ In vivo imaging provided evidence that cellular and tracer transport between CSF and skull bone marrow in the dura-skull channels occur along perivascular spaces of the bridging vessels.^12,23^ One study observed meningeal B-cell migration through skull vascular channels from the calvaria to the meninges.^11^ Another investigation showed neutrophil migration through channels connecting the skull bone marrow with the dura.^21^ A third study reported myeloid cell infiltration from skull bone marrow to brain meninges and brain parenchyma during CNS injury through skull-dural channels.^10^ While not demonstrated, these authors speculated that myelomonocytic cells pass along vessels residing within channels connecting skull bone marrow with dura.

The methods used to study the passage of substances between the CSF and skull bone marrow included injections of tracers into the CSF^12,22,23,25^ and administering bacteria to the cisterna magna.^23^ A common tracer is fluorescent-tagged ovalbumin, which has a molecular weight about 45 kDa. Moreover, mouse models of brain disease or injury^10,19,21,22^ were applied to study the migration of leukocytes from skull bone marrow to the meninges and/or brain parenchyma. Another approach was in vivo cell labeling of bone marrow cells in the mouse skull and confocal plus electron microscopy of channels connecting the skull and dura.^21^ Two studies performed histological cross-sections of the mice brain with adjacent skull bone.^20,24^ Specific nano-labeling was used to enhance signal of fluorescent proteins, thereby allowing a high degree of transparency through the mouse skull and the opportunity to visualize subcellular details, including vascular channels between the skull and brain meninges.^22^

In summary, several studies provide evidence for channels between the dura mater and skull bone marrow that harbor vessels where the bony perivascular space may serve as a conduit for cells and solutes.

### Functional evidence

Studies examining the functional aspects of crosstalk between brain, CSF, meninges and skull explored the transport of molecules and cells. Srebro et al.^24^ reported that H-thymidine, injected intrathecally in mice, penetrated into the sphenoid bone marrow from the arachnoid space and labeled leukocytes in the skull bone marrow.^24^ More recently, two studies^12,23^ showed that a CSF tracer administered to the cisterna magna passed into the skull bone marrow. Administering a tracer to the CSF revealed tracer uptake in meningeal lymphatic vessels that penetrated the skull through bony openings.^25^ On the other hand, external application of manganese chloride (MnCl_2_) to an exposed skull bone of rats, showed diffusion of manganese (Mn^2+^) ions through the skull bone into the brain, detectable by MRI.^26^ However, this study did not define the anatomical route other than that the manganese ions penetrates the meninges to enter the cerebral cortex.

Several studies reported the exchange of cells between CSF and the skull bone at the meninges. Six studies observed myeloid and lymphoid cell migration from the skull bone marrow to the brain meninges in mice, including meningeal B-lymphocytes,^11,27^ T-lymphocytes,^27^ monocytes,^10,12^ macrophages and neutrophils.^12^ Cugurra et al.^10^ provided evidence that the skull bone marrow supplies monocytes to the meninges and brain parenchyma both in health and disease. They reported that following brain injury, monocytes derived from skull bone marrow infiltrate the brain through channels in skull bone marrow and dura. In another study, meningeal B-cells migrate from the calvaria to the meninges through vascular channels.^11^ In addition, neutrophil cells were found to migrate through direct vascular channels, which cross the inner skull bone, connecting the skull bone marrow spaces with the dura mater;^21^ however, passage of cells between dura mater and subarachnoid space was not addressed. Okonogi et al.^28^ reported that cranial irradiation could induce bone marrow derived microglia (BMDM) in adult mouse brain. The exact passage route was not defined in this study, and the authors speculated about passage via a disrupted blood-brain-barrier. One of the included articles reported migration of bacteria (streptococcus pneumonia) from the meninges to the skull bone marrow after the injection of bacteria in the cisterna magna of mice.^23^

Yao et al.^20^ reported migration of acute lymphoblastic leukemia (ALL) cells into the brain along vessels passing directly between the skull bone marrow and the subarachnoid space. The vessels were found to cross through bony channels of the skull, connecting dura-skull bone, enabling direct communication between skull bone marrow and the subarachnoid space.^20^ It is well established that ALL cells fail to breach the blood-brain-barrier (BBB), and that metastasis from ALL seldom involve the brain parenchyma but restricted to the leptomeninges. It is therefore notable that the basement membrane of the bridging vessels was found rich in laminin, and that the perivascular passage of cells was mediated by α6 integrin-laminin interactions. This could become a therapeutic target since most ALL cases express laminin receptor α6 integrin and blockade of this receptor was found to inhibit the perivascular tumor cell transport.

Another approach to study the possible therapeutic potential of connections between skull bone marrow and CSF of the subarachnoid space was explored by Kang et al.^29^ who administered nine different drugs within the skull bone marrow (intracalvariosseous), measured brain concentrations and performed quantitative pharmacokinetic analysis. The nine tested drugs gave substantially higher brain concentrations (minimum 2-fold to maximum 342-fold) than the corresponding intravenous administration. The authors concluded that the transport route was extravascular rather than intravascular via vascular channels/connections between the skull bone marrow and the meninges.^29^

### Human studies

#### Anatomical evidence

Four of the human studies provided anatomical evidence for a connection between the skull bone marrow and the meninges or subarachnoid space^21,25,27,30^ (Table 2). Tsutumi et al.^30^ observed CSF filled channels continuing with the subarachnoid spaces in the middle fossa, and Herrison et al.^21^ identified in both mice and humans skull channels crossing the inner skull cortex from the skull bone marrow to the dural meninges. Jacob et al.^25^ observed what they interpreted as perforating meningeal lymphatic vessels and slow-flow channels crossing the skull, thus providing a link between the subarachnoid space, dura and calvarial bone. A recent study characterized skull-meninges-connections at the cellular level, bridging the dura mater with the skull bone marrow. The study was conducted on human skull-dura postmortem samples. They observed that the skull-meninges-connections transverse the dura mater, opening to the subdural space underneath to arachnoid granulations in humans.^27^ These authors quantified over 500 skull-meningeal connections, and found that they are 40–90 µm wide, and occasionally >150 µm wide. They further found that PDGFR-B signal was present at both the vessels and the skull-meningeal connection lumen, indicating that the lumen of the skull-meningeal connections is lined by fibroblastic cells that are known antigen-presenting cells. In addition to the fibroblastic cell layer, immune cells were present.

#### Functional evidence

Four human studies provided functional evidence of passage between the skull bone and the subarachnoid space; three studies examining tracer transport,^15,25,31^ and one revealing channels harboring cells^27^ (Table 2). These studies primarily provide evidence for transport of solutes.

The first study to demonstrate efflux of CSF into skull bone marrow of any species utilized an MRI contrast agent as CSF tracer, enabling visualization of tracer enrichment between subarachnoid CSF and the skull bone marrow across the meninges in a patient group.^15^ Another study examined upregulation of an inflammatory marker in meninges and skull bone in human patients with migraine with visual aura. Using ^11^C-PBR28 PET/MRI with the PET ligand ^11^C-PBR28 serving as an inflammatory marker, they found that migraine patients with aura had significantly higher tracer uptake (i.e. increased inflammatory signal) in the meninges and the adjacent skull bone overlying occipital cortex than control groups.^31^ The authors speculated that the meningeal/calvarial inflammation was caused by direct connections between the cerebral cortex and meninges and skull bone, but the study did not address the substances or passage route involved.

## Discussion

This systematic literature review revealed studies providing evidence for 1) anatomic bony channels between the dura and the skull bone marrow, 2) blood vessels residing within these bony channels linking the subarachnoid space with the skull bone marrow, 3) passage of cells and solutes along the outside of these vessels, and 4) evidence for bidirectional transport, for example, CSF moving from subarachnoid space to the skull bone marrow and cells moving from the skull bone marrow to the subarachnoid space. Most data are derived from animal experiments, emphasizing the need for confirmation in humans.

The research field is young, as most of the literature has emerged since 2015. An exception is the 1993 study by Srebro et al.,^24^ providing the first evidence of direct vascular connections between skull bone and the brain.

## Transport capacity

An unresolved question is how much cells and solutes exchange between subarachnoid CSF spaces, dura and skull bone marrow – that is, what is the transport capacity? This likely depends on the region. Conversely, considering that CSF is continuously presented to the skull bone marrow and possesses the ability to instruct cellular responses,^12^ the skull bone marrow may play a pivotal role in regulating CNS immune responses. Consequently, previously the importance of CSF as a messenger to instruct inflammatory responses of the CNS seem to have been underestimated. CSF may thus be essential for CNS immunosurveillance and waste clearance. In this regard, waste clearance via meningeal lymphatic structures may be of importance in Alzheimer’s disease (clearance of amyloid-β and tau)^32–34^ and in Parkinson’s disease (clearance of α-synuclein).^35,36^

Concerning translation to humans, a major outstanding question revolves around methods for visualizing the passage of substances across the brain borders. Currently, the use of MRI to identify lymphatic vessels in human dura mater remains highly debated.^37–40^ Caution is recommended to avoid over-interpretation of structures interpreted as “lymphatic vessels” at MRI,^25^ as they may instead represent various types of structures in the dura, such as veins, a dense network of capillaries, liquid lakes, intradural arachnoid granulations, and dural stroma.^41,42^

## Efflux routes

A critical inquiry pertains to how cells and solutes traverse the arachnoid, given prior evidence of arachnoid barrier cells forming an impermeable obstacle to substances within the subarachnoid CSF.^4^ Smyth et al.^43^ recently identified gaps in the arachnoid barrier cell layer where bridging veins from cerebral cortex enter the dura mater. These structures, termed arachnoid cuff exits (ACE), encircle veins that penetrate the arachnoid barrier cell layer, providing a conduit for cells and substances between subarachnoid CSF and the dura mater. Notably, arachnoid border cells were labeled by Prox1-eGFP, which also marked the so- called subarachnoid lymphatic-like membrane (SLYM), posited as a fourth brain meninges alongside the pia mater, arachnoid mater and dura mater.^3^ Prox1 was also recently found to label a fibroblast layer within the subarachnoid space,^4,44^ though conflicting evidence has been provided regarding the existence of the SLYM and a compartmentalized subarachnoid space.^3,4^ On the other hand, the hypothesis of a non-compartmentalized subarachnoid space^4^ is challenged by human in vivo observations of a perivascular subarachnoid spaces (PVSAS) that facilitates the passage of tracer within the subarachnoid space and along the arteries toward the brain.^6^

**Figure 4.**
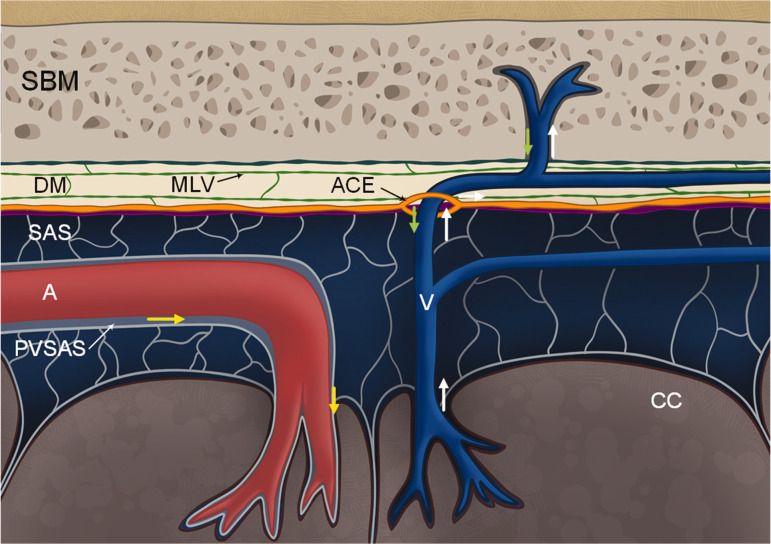
Cartoon illustrating current evidence derived from the literature review regarding the potential route for passage of cells and solutes from subarachnoid CSF space to skull bone marrow across meninges. The review highlights numerous studies demonstrating the existence of vascularized bone channels in the skull bone, extending from the skull bone marrow (SBM) to the dura mater (DM).^10–12,19–24,27^ Blood vessels within these bony channels traverse the dura mater and extend across the arachnoid membrane to reach the subarachnoid space (SAS).^20,23^ Recent findings revealed the presence of arachnoid cuff exits (ACE) forming cuffs around vessels,^43^ enabling the passage of cells and substances along the arachnoid barrier cells adjacent to the dura border cells. Research indicates that the basement membrane of vessels serves as a transport pathway for cells and substances.^20,43^ This basement membrane is notably rich in laminin, which may interact with α6 integrin, enabling transport of ALL cells from skull bone marrow to the subarachnoid space.^20^ Remarkably, molecules of diverse mass and properties, including dextrans with a molecular weight of 70,000 kDa, can traverse this pathway.^43^ Within the dura mater, meningeal lymphatic vessels are present, and notably, there is no blood-brain-barrier within the dura. It is further indicated that CSF influx (yellow arrows) occurs along perivascular subarachnoid space (PVSAS)^6^ towards cerebral cortex (CC), CSF efflux (white arrows) along veins, and via ACE to dura and skull bone marrow. From skull bone marrow cells and substances (green arrow) may pass along vessels within bone channels, across dura to the subarachnoid space. Illustration: Øystein Horgmo, University of Oslo.

Emerging evidence indicates that substances within CSF may traverse along vessels from the subarachnoid space to the dura mater. The molecular composition of the abluminal part of vessels, currently emphasizing laminin and α6 integrin interactions, appears crucial for the perivascular passage of cells and substances from CSF towards the dura and skull bone marrow (refer to Figure 4). α6 integrin-laminin interactions for abluminal passage of ALL cells between the skull bone marrow and subarachnoid CSF.^20^ In this regard, it is noteworthy that laminin staining was intense around the ACE points,^43^ and the authors proposed that laminin and α6 integrin interactions are pivotal for trafficking cells, including myeloid cells, to the subarachnoid space at ACE points. This perspective aligns with observations of intrathecal CSF tracer passing from the subarachnoid space to the dura along vessels (perivascular), through skull bone channels to skull bone marrow cavities.^23^

Diverse molecules and cells have been shown to pass between brain and skull bone marrow across meninges. These include MRI contrast agents (e.g., gadobutrol, molecular weight 604 Da),^15^ large-molecular size compounds such as ovalbumin (45k Da),^23,25^ dextrans (up to 70,000 kDa),^23^ and smaller sized substances like manganese ions (molecular weight about 55 Da),^26^ which is comparable to water in size, and H-thymidine (molecular weight about 242 Da).^24^ The various cell types studied include myeloid cells,^10,12^ meningeal B-cells^11^ (only from skull bone marrow to the meninges), neutrophils^21^ (migrating through channels from skull bone marrow to the dura), macrophages (only from meninges to brain).^19^ One study observed bacterial cell migration to the skull bone from brain,^23^ and passage of tumor or leukemia cells.^20,24^ With regard to the recently discovered ACE points, substances of variable molecular masses and properties (including dextrans of 10, 70 and 2,000 kDa, ovalbumin 45 kDa, and 0.5 µm polystyrene beads) passed via these points, indicating bulk flow properties.^43^

## Understanding and treatment of neurological diseases

Another question is the implications the new knowledge may have for understanding and treating neurological diseases. Since immune cells and substances can be released directly from the skull bone marrow and dura into the CSF, skull bone marrow-derived cells and molecules may participate in brain immune surveillance at the meninges and also be carried into the brain.^15^ Additionally, communication between the intracranial compartment and bone marrow may enhance our understanding of common symptoms such as headache during influenza – since viruses can directly infect bone marrow, and now can be hypothesized to inflict an associated inflammation in adjacent meninges or periosteum.^15^ Moreover, Hadjikhani et al.^31^ showed an elevated inflammatory signal in the overlying meninges and skull bone of the occipital lobe in migraine patients with aura, suggesting that meningeal inflammation contributes to the pathophysiology of photophobia. The authors proposed that the inflammatory signal migrates from the underlying brain to the meninges and bone marrow, indicating cross talk between bone marrow and brain.^31^ The skull bone marrow may function as a hub for immune progenitor cells, which, upon contact with antigens from bacteria, may develop, proliferate, and migrate to the meninges and brain parenchyma to perform their functions during infections or brain injuries. A direct pathway may have great significance in neural inflammation. The skull bone channels may function as ducts for bacteria, antigen, and cytokine migration into the skull bone marrow, which then can induce proliferation of white blood cells in the calvarial bone marrow.^23^ Additionally, myeloid cells originating from the local skull bone marrow rather than from the blood may serve as a “myeloid” reservoir for meninges and brain. Experimental evidence indicates that CSF may instruct cell activation within the bone marrow, allowing for subsequent entry of leukocytes in the dura and brain tissue during brain infections or after CNS injury.^12^

The study conducted by Kolabas et al.^27^ observed skull inflammation subsequent to brain inflammation in patients with neurological diseases. They identified a disease-specific increase in TSPO-PET-signal in calvarial regions in patients with Alzheimer’s disease and other neurological diseases. TSPO is a protein significantly upregulated in the brain during neuroinflammation, showing a strong correlation between changes in the skull and brain translocator protein-PET signal in patient with Alzheimer’s disease. These novel findings may establish a link between the skull and neurological diseases in humans, suggesting that TSPO-PET imaging of the skull can reveal distinct signal patterns in inflammatory and degenerative CNS conditions. Could it be that these skull-brain communication channels play an important role in neurodegenerative diseases including Alzheimer’s disease? And could it be that the bone marrow of skull could serve as a novel target in diagnosis of brain diseases?

Another aspect emerging from this review is the potential for future therapeutic approaches. For instance, the study documenting manganese diffusion from the skull into the brain^26^ suggests a novel and less invasive route for drug delivery to the brain. A recent study hypothesized that the direct vascular channels between the skull bone marrow and the meninges may also enable drug delivery from the skull bone marrow to the brain.^29^ The study reported that selected compounds reached the brain tissue several times higher by intracalvariosseus administration than by systemic application. These findings suggest that administration of CNS drugs into the skull bone could be a new pathway or approach for effective treatment of brain diseases. Moreover, the study conducted in migraine patients with aura, showing an enhanced inflammation signal in the skull bone overlying the occipital cortex,^31^ might suggest alternative treatment options for migraine and neuro-inflammatory diseases. Additionally, the findings of a direct communication between bone marrow and CSF enlighten our understanding of the mechanisms underlying autoimmune and neurodegenerative brain disorders and may lead to the development of new therapeutic approaches for diseases in the central nervous system.

## Outlook and conclusions

While our primary focus in this review is the interaction between the brain and the skull bone marrow, it is important to note that the vertebral bone marrow also plays a significant role in influencing the spinal cord. Given that CSF circulates throughout the entire craniospinal compartment, this interplay not only affects the brain but also the entire CNS. A recent review highlights that spinal cord trauma in mice results in alterations in CSF composition, which subsequently affects the adjacent vertebral bone marrow.^45^ In essence, it appears that CSF carries signals to the vertebral bone marrow, stimulating myelopoiesis and supplying cells to the underlying meninges and spinal cord. These findings offer functional evidence for the interaction between vertebral bone marrow and the spinal cord, although further research is warranted to explore the anatomical evidence of this interaction.From the present literature review, a picture emerges that the brain borders, including the meninges and skull bone marrow, play an essential role in both immune surveillance and waste clearance. Instead of being regarded as separate anatomical entities, the CNS-meninges-skull organ-system should rather be viewed as an anatomical and functional continuum. Since an organ is defined as a collection of specialized tissues that structurally form a functional unit, specialized to perform a particular function, the brain meninges may in this respect by definition be considered as a separate organ.

Taken together, emerging evidence from the last decade has demonstrated existence of vascular bone channels extending from the skull bone marrow to the dura mater. These channels may host vessels that bridge the previously considered impermeable barriers, including meninges, between the brain, subarachnoid space and the skull bone marrow. Recent research has revealed the existence of cuffs or openings around bridging veins, facilitating the passage of even large mass substances from the subarachnoid space to the dura mater. Current evidence suggests that the transport route occurs along the vessel (abluminal), specifically at the basement membrane, where the molecular composition is crucial. This emphasizes the significant role of the abundant expression of laminin within the basement membrane and its interactions with α6 integrin. While several questions persist, the brain meninges now emerge as a specialized structure with distinct functions, particularly in waste clearance and central nervous system (CNS) immunosurveillance.

## Supplemental Material

sj-pdf-1-jcb-10.1177_0271678X251316392 - Supplemental material for Evidence for cellular and solute passage between the brain and skull bone marrow across meninges: A systematic review Supplemental material, sj-pdf-1-jcb-10.1177_0271678X251316392 for Evidence for cellular and solute passage between the brain and skull bone marrow across meninges: A systematic review by Helena Eide Therkelsen, Rune Enger, Per Kristian Eide and Geir Ringstad in Journal of Cerebral Blood Flow & Metabolism
