# Supplementary material for: Evidence for cellular and solute passage between the brain and skull bone marrow across meninges: A systematic review
Source: J Cereb Blood Flow Metab. 2025 Jan 25;45(4):581–99. doi: 10.1177/0271678X251316392 (PMC11765306; doi:10.1177/0271678X251316392)
Supplement: sj-pdf-1-jcb-10.1177_0271678X251316392 - Supplemental material for Evidence for cellular and solute passage between the brain and skull bone marrow across meninges: A systematic review [file sj-pdf-1-jcb-10.1177_0271678X251316392.pdf]

Supplementary Material

Evidence for cellular and solute passage between the brain and skull bone marrow across meninges: A systematic review

Helena Eide Therkelsen[1], Rune Enger[2,3,4], Per Kristian Eide[3,4,5], Geir Ringstad[3,5,6,7]

[1]*Faculty of Medicine, University of Oslo, Oslo, Norway*

[2]*Institute of Basic Medical Sciences, Department of Molecular Medicine, University of Oslo, Norway,*

[3]*KG Jebsen Centre for Brain Fluid Research, University of Oslo, Oslo, Norway,*

[4]*Deptartment of Neurosurgery, Oslo University Hospital-Rikshospitalet, Oslo, Norway,*

[5]*Institute of Clinical Medicine, Faculty of Medicine, University of Oslo, Oslo, Norway,*

[6]*Department. of Radiology, Oslo University Hospital-Rikshospitalet, Oslo, Norway.*

[7]*Dept. of Geriatrics and Internal medicine, Sorlandet Hospital, Arendal, Norway*

Corresponding author:
Professor Per Kristian Eide, MD PhD
Department of Neurosurgery
Oslo University Hospital - Rikshospitalet
Pb 4950 Nydalen,
N-0424 Oslo, Norway
p.k.eide@medisin.uio.no

Content

Supplementary Table 1. Systematic Review Search Protocol

Supplementary Table 2. References

Supplementary Table 1. Systematic Review Search Protocol

Sources used for the search:

Database	January 11th 2024
MEDLINE (Ovid):	388
Embase (Ovid):	520
Cochrane Library:	290
Number of references prior to duplicate deletion	1198
Number of references after duplicate deletion	663

The search was initially performed on January 6th 2022 and updated on January 11th 2024. The search was conducted by Helena Eide Therkelsen and Hilde Iren Flaatten, Medical Librarian, University of Oslo: Library of Medicine and Science. Literature searching and Evidence-Based Practice Unit

---

Database: Medline

1. Cerebrospinal Fluid/ or (((cerebrospinal or cerebro spinal or ventricle*) adj2 (fluid* or liquor*)) or CSF).tw,kf.

2. meninges/ or arachnoid/ or subarachnoid space/ or dura mater/ or subdural space/ or pia mater/

3. (meninge* or meninx* or arachnoid* or subarachnoid* or dura or dural or subdural or pia mater* or brain covering*).tw,kf.

4. (((brain* or cerebral) adj3 (surface* or tissue*)) or CNS border*).tw,kf.

5. or/1-4

6. exp skull/ or (skull* or scull* or diploe* or diploic or intradiplo* or cranium or calvaria or calvarium or ((cranial or temporal or frontal or occipital or parietal) adj bone*)).tw,kf.

7. (glymphatic or crosstalk* or cross-talk* or ((vascular or intradural) adj2 channel*) or ((ion* or molecul* or microbe* or micromolecul* or macromolecul* or cell* or protein* or leukocyte* or t cell* or monocyte* or neutrophil* or eosinophil* or basophil* or macrophage* or lymphocyte* or immun* or myeloid*) adj5 (path* or invad* or channel* or migrat* or transmigrat* or rolling or motilit* or pass* or motion* or mov* or flow* or transport* or transfer* or clearance* or exchang* or diffus* or connect* or bypass* or interact* or spread* or homing or locomotion* or traffic* or infiltrat*))).tw,kf.

8. biological transport/ or exp biological transport, active/ or ion transport/ or exp protein transport/ or exp Cell Movement/ or Glymphatic System/

9. 7 or 8

10. 5 and 6 and 9

11. limit 10 to english language

Database: Embase

1. cerebrospinal fluid/ or (((cerebrospinal or cerebro spinal or ventricle*) adj2 (fluid* or liquor*)) or CSF).tw,kf.

2. exp meninx/ or (meninge* or meninx* or arachnoid* or subarachnoid* or dura or dural or subdural or pia mater* or brain covering*).tw,kf.

3. (((brain* or cerebral) adj3 (surface* or tissue*)) or CNS border*).tw,kf.

4. 1 or 2 or 3

5. skull/ or calvaria/ or skull base/ or (skull* or scull* or diploe* or diploic or intradiplo* or cranium or calvaria or calvarium or ((cranial or temporal or frontal or occipital or parietal) adj bone*)).tw,kf.

6. (glymphatic or crosstalk* or cross-talk* or ((vascular or intradural) adj2 channel*) or ((ion* or molecul* or microbe* or micromolecul* or macromolecul* or cell* or protein* or leukocyte* or t cell* or monocyte* or neutrophil* or eosinophil* or basophil* or macrophage* or lymphocyte* or immun* or myeloid*) adj5 (path* or invad* or channel* or migrat* or transmigrat* or rolling or motilit* or pass* or motion* or mov* or flow* or transport* or transfer* or clearance* or exchang* or diffus* or connect* or bypass* or interact* or spread* or homing or locomotion* or traffick* or infiltrat*))).tw,kf.

7. exp transport at the cellular level/ or cell motion/ or exp cell migration/ or exp cell motility/ or glymphatic system/

8. 6 or 7

9. 4 and 5 and 8

10. limit 9 to conference abstracts

11. 9 not 10

12. limit 11 to english language

Database: Cochrane Library, Web of Science

#1 AND #2 AND #9 and English (Languages)

#1 TS=(((skull* or scull* or diploe* or diploic or intradiplo* or cranium or calvaria or calvarium or ((cranial or temporal or frontal or occipital or parietal) NEXT bone*))))

#2 ((TS=((((cerebrospinal or "cerebro spinal" or ventricle*) NEAR/1 (fluid* or liquor*)) or CSF))) OR TS=((meninge* or meninx* or arachnoid* or subarachnoid* or dura or dural or subdural or pia mater* or brain covering*))) OR TS=((((brain* or cerebral) NEAR/2 (surface* or tissue*)) or CNS border*))

#3 OR #4 OR #5 OR #6 OR #7 OR #8

TS=(((immun* or myeloid* or monocyte* or neutrophil* or eosinophil*) NEAR/4 (path* or invad* or channel* or migrat* or transmigrat* or rolling or motilit* or pass* or motion* or mov* or flow* or transport* or transfer* or clearance* or exchang* or diffus* or connect* or bypass* or interact* or spread* or homing or locomotion* or traffick* or infiltrat*)))

TS=(((basophil* or macrophage* or lymphocyte*) NEAR/4 (path* or invad* or channel* or migrat* or transmigrat* or rolling or motilit* or pass* or motion* or mov* or flow* or transport* or transfer* or clearance* or exchang* or diffus* or connect* or bypass* or interact* or spread* or homing or locomotion* or traffick* or infiltrat*)))

TS=(((leukocyte* or "t cell*") NEAR/4 (path* or invad* or channel* or migrat* or transmigrat* or rolling or motilit* or pass* or motion* or mov* or flow* or transport* or transfer* or clearance* or exchang* or diffus* or connect* or bypass* or interact* or spread* or homing or locomotion* or traffick* or infiltrat*)))

TS=(((micromolecul* or macromolecul* or cell* or protein*) NEAR/4 (path* or invad* or channel* or migrat* or transmigrat* or rolling or motilit* or pass* or motion* or mov* or flow* or transport* or transfer* or clearance* or exchang* or diffus* or connect* or bypass* or interact* or spread* or homing or locomotion* or traffick* or infiltrat*)))

TS=(((ion* or molecul* or microbe*) NEAR/4 (path* or invad* or channel* or migrat* or transmigrat* or rolling or motilit* or pass* or motion* or mov* or flow* or transport* or transfer* or clearance* or exchang* or diffus* or connect* or bypass* or interact* or spread* or homing or locomotion* or traffick* or infiltrat*)))

TS=((glymphatic or crosstalk* or cross-talk* OR ((vascular or intradural) NEAR/1 channel*)))

Supplementary Table 2. References

1. Srebro Z, Pierscinska E, Plonkowa I. Communication of the intracranial space with sphenoid bone marrow in mice. Folia Biol (Krakow). 1993;41(1-2):25-7.
2. Atanasijevic T, Bouraoud N, McGavern DB, Koretsky AP. Transcranial manganese delivery for neuronal tract tracing using MEMRI. Neuroimage. 2017;156:146-54.
3. Herisson F, Frodermann V, Courties G, Rohde D, Sun Y, Vandoorne K, et al. Direct vascular channels connect skull bone marrow and the brain surface enabling myeloid cell migration. Nat Neurosci. 2018;21(9):1209-17.
4. Yao H, Price TT, Cantelli G, Ngo B, Warner MJ, Olivere L, et al. Leukaemia hijacks a neural mechanism to invade the central nervous system. Nature. 2018;560(7716):55-60.
5. Cai R, Pan C, Ghasemigharagoz A, Todorov MI, Förstera B, Zhao S, et al. Panoptic imaging of transparent mice reveals whole-body neuronal projections and skull-meninges connections. Nat Neurosci. 2019;22(2):317-27.
6. Tsutsumi S, Ono H, Yasumoto Y, Ishii H. Possible cerebrospinal fluid pathways in the middle fossa floor and pterional diploe: a magnetic resonance imaging study. Surg Radiol Anat. 2019;41(9):1045-51
7. Wang F, Wan H, Ma Z, Zhong Y, Sun Q, Tian Y, et al. Light-sheet microscopy in the near-infrared II window. Nat Methods. 2019;16(6):545-52.
8. Hadjikhani N, Albrecht DS, Mainero C, Ichijo E, Ward N, Granziera C, et al. Extra-Axial Inflammatory Signal in Parameninges in Migraine with Visual Aura. Ann Neurol. 2020;87(6):939-49.
9. Brioschi S, Wang WL, Peng V, Wang M, Shchukina I, Greenberg ZJ, et al. Heterogeneity of meningeal B cells reveals a lymphopoietic niche at the CNS borders. Science. 2021;373(6553) (no pagination).
10. Cugurra A, Mamuladze T, Rustenhoven J, Dykstra T, Beroshvili G, Greenberg ZJ, et al. Skull and vertebral bone marrow are myeloid cell reservoirs for the meninges and CNS parenchyma. Science. 2021;373(6553) (no pagination).
11. Jacob L, Neto JDB, Lenck S, Corcy C, Benbelkacem F, Geraldo LH, et al. Conserved meningeal lymphatic drainage circuits in mice and humans. J Exp Med. 2022;219(8):27.
12. Mazzitelli JA, Smyth LCD, Cross KA, Dykstra T, Sun J, Du SL, et al. Cerebrospinal fluid regulates skull bone marrow niches via direct access through dural channels. Nat Neurosci. 2022;25(5):555-+.
13. Pulous FE, Cruz-Hernandez JC, Yang CB, Kaya Z, Paccalet A, Wojtkiewicz G, et al. Cerebrospinal fluid can exit into the skull bone marrow and instruct cranial hematopoiesis in mice with bacterial meningitis. Nat Neurosci. 2022;25(5):567-+.
14. Ringstad G, Eide PK. Molecular trans-dural efflux to skull bone marrow in humans with CSF disorders. Brain. 2022;145(4):1464-72.
15. Kang JH, Ko YT. Intraosseous administration into the skull: Potential blood-brain barrier bypassing route for brain drug delivery. Bioeng Transl Med. 2023;8(2):10.
16. Kolabas ZI, Kuemmerle LB, Perneczky R, Forstera B, Ulukaya S, Ali M, et al. Distinct molecular profiles of skull bone marrow in health and neurological disorders. Cell. 2023;186(17):3706-25.e29.
